# Effects of Baduanjin on postoperative rehabilitation of patients with breast cancer: A protocol for systematic review and meta-analysis

**DOI:** 10.1097/MD.0000000000025670

**Published:** 2021-04-30

**Authors:** Guangyan Chen, Yuqin Lin, Xiyun Zhao, Bin Pu

**Affiliations:** a Traditional Medical Diagnosis and Treatment Centre, Gansu Provincial People's Hospital, Lanzhou; b Wuwei Hospital of Traditional Chinese Medicine, Wuwei; c Intervertebral Disc Center, Affiliated Hospital of Gansu University of Traditional Chinese Medicine, Lanzhou, China.

**Keywords:** Baduanjin, breast cancer, effect, rehabilitation, systematic review

## Abstract

**Background:**

Baduanjin, as an ancient Chinese exercise, is beneficial to both physical and mental health. Moreover, researchers discovered that Baduanjin has effects on the recovery of postoperative breast cancer patients. Yet, nobody focused on the systematic review, which can provide convincing evidence to verify the effect of Baduanjin in breast cancer patients. Therefore, our study will conduct a systematic review to fill in the blank, besides we will offer new evidence for clinical workers.

**Methods:**

PubMed, Embase.com, the Cochrane Central Register of Controlled Trials (CENTRAL), Web of Science, China National Knowledge Infrastructure (CNKI), Wanfang, and SinoMed will be used for literature search, retrieve time is up to June 1, 2021. We will include randomized controlled trials that evaluate the effects of Baduanjin on postoperative rehabilitation for breast cancer patients. Two independent researchers will perform study selection and data extraction. The risk of bias will be assessed by the Cochrane bias assessment tool. We will use funnel plot and Egger test to evaluate publication bias. Stata 13.0, as a necessary software, will be used to perform statistical analysis. Also, we will utilize subgroup analyses and sensitivity analyses to explore the sources of heterogeneity.

**Results:**

The results of this study will be published in a peer-reviewed journal.

**Conclusion:**

Evidence that adequately assesses the effect of Baduanjin in the recovery of breast cancer patients will be confirmed through this systematic review. Our study will offer a guideline for clinical workers, besides we will supply a new way for the rehabilitation of breast cancer patients.

## 1 Introduction

In recent years, breast cancer has become the most common cancer in women.^[1]^ According to the data published in 2019, about 62930 cases of female breast carcinoma are expected to diagnose, which almost accounts for 30% of the newly diagnosed cancer of women.^[2]^ In China, breast cancer has been the leading cause of cancer death among women, next to lung cancer. Furthermore, there is a rising trend in the age-standardized incidence rate for women.^[3]^ Aging, family, history, reproductive factors, estrogen, and lifestyle have become the primary risk factors of breast cancer. Especially relevant to increasing age, up to 83% of all breast cancer-associated death was reported in women over the age of 50.^[4,5]^ More recently, options on breast cancer treatment, such as surgery, chemotherapy, hormone replacement therapy, radiation therapy, complementary therapy developed rapidly.^[6]^ Thus, the attention of clinicians was switched to rehabilitation while more and more patients got relief from these regular therapies.^[7]^ The key aspects of recovery programs can be listed as follows: daily exercise (Baduanjin, Taijiquan), psychotherapy, education, and rehabilitation nursing.

Baduanjin, a traditional exercise (a type of Qigong) with a history of over 2000 years, is composed of 8 sections, they are support heaven with hands, dragon sprays water with force, big bird spreads its wings, lift window to look at the moon on the left, descend to earth with force, beautiful maiden twists her waist to the right, extend’ shoulders to bring hands together, dragon claws to the left.^[8]^ As an ancient beneficial exercise, Baduanjin not only strengthens our fitness, such as physical flexibility and subcutaneous adipose accumulation, sleeping qualities, relief in low back pain but also improves our mental health.^[9–12]^ Compared with regulation treatments, Baduanjin is an economic exercise and very easy to learn. Moreover, Baduanjin has shown positive impacts on heart failure, knee osteoarthritis, diabetes mellitus, and postoperation rehabilitation.^[13–15]^ For these reasons, studies about the effect of Baduanjin in the recovery of breast cancer patients begin to cause comprehensive concern.^[16]^ Yet, much of these researches up to now have not focused on the systematic review, which can provide convincing evidence to verify the effect of Baduanjin in breast cancer patients. Thus, the purpose of our study is to fill in the blank, meanwhile, we will also provide new evidence for clinical workers.

## 2 Methods

### 2.1 Registration

This systematic review protocol has been registered on the International Platform of Registered Systematic Review and Meta-analysis Protocols (INPLASY), registration number is INPLASY202140018. This systematic review protocol is conducted and reported according to the Preferred Reported Items for Systematic Reviews and Meta-Analyses Protocols guidelines.^[17]^

### 2.2 Search strategy

Experienced researchers will develop a comprehensive search strategy. The literature search will be conducted on the following database: PubMed, Embase. com, the Cochrane Central Register of Controlled Trials (CENTRAL), Web of Science, China National Knowledge Infrastructure (CNKI), Wan Fang, and SinoMed. Retrieve time is from the establishment of the databases to June 1, 2021. We will also manually search the references of related articles. Take PubMed as an example, search strategy is shown in Table 1.

**Table 1:** Search strategy of PubMed.

Stage	Search strategy
#1	“Breast Neoplasms” [Mesh] OR “Breast Carcinoma In Situ” [Mesh] OR “Breast Neoplasms, Male” [Mesh] OR “Carcinoma, Ductal, Breast” [Mesh] OR “Carcinoma, Lobular” [Mesh] OR “Inflammatory Breast Neoplasms” [Mesh] OR “Triple Negative Breast Neoplasms” [Mesh] OR “Unilateral Breast Neoplasms” [Mesh] OR breast neoplasm∗ [Title/Abstract] OR breast tumor∗ [Title/Abstract] OR breast carcinoma∗ [Title/Abstract] OR breast cancer∗ [Title/Abstract] OR breast tumour∗ [Title/Abstract] OR mammary neoplasm∗ [Title/Abstract] OR mammary tumor∗ [Title/Abstract] OR mammary carcinoma∗ [Title/Abstract] OR mammary cancer∗[Title/Abstract] OR mammary tumour∗ [Title/Abstract] OR breast adenocarcinoma∗ [Title/Abstract] OR breast carcinogenesis [Title/Abstract] OR breast sarcoma∗ [Title/Abstract] OR phyllodes tumor∗ [Title/Abstract] OR intraductal carcinoma∗ [Title/Abstract] OR lobular carcinoma∗ [Title/Abstract]
#2	Baduanjin [Title/Abstract] OR Baduan jin [Title/Abstract] OR Baduanjin exercise∗ [Title/Abstract] OR BDJ [Title/Abstract] OR eight section brocade∗ [Title/Abstract] OR eight-section Brocade∗ [Title/Abstract] OR eight trigrams boxing [Title/Abstract] OR eight-treasured exercise∗ [Title/Abstract] OR eight pieces of brocade∗ [Title/Abstract] OR eight brocade section [Title/Abstract] OR Chinese regimen∗ [Title/Abstract] OR Chinese ancient regimen∗ [Title/Abstract] OR “qigong” [Mesh] OR qigong [Title/Abstract] OR Qi Gong [Title/Abstract] OR Chikung∗ [Title/Abstract] OR “rehabilitation exercise” [Mesh] OR rehabilitation exercise∗ [Title/Abstract] OR Remedial Exercise∗ [Title/Abstract] OR Exercise, Remedial∗ [Title/Abstract] OR Exercises, Remedial∗ [Title/Abstract] OR Remedial Exercises∗ [Title/Abstract] OR Therapy, Exercise∗ [Title/Abstract] OR Exercise Therapies∗ [Title/Abstract] OR Therapies, Exercise∗ [Title/Abstract] OR Rehabilitation Exercise∗ [Title/Abstract] OR Exercise, Rehabilitation∗ [Title/Abstract] OR Exercises, Rehabilitation∗ [Title/Abstract] OR Rehabilitation Exercises∗ [Title/Abstract]
#3	“Clinical Trials, Phase II as Topic” [Mesh] OR “Clinical Trials, Phase III as Topic” [Mesh] OR “Clinical Trials, Phase IV as Topic” [Mesh] OR “Controlled Clinical Trials as Topic” [Mesh] OR “Randomized Controlled Trials as Topic” [Mesh] OR “Intention to Treat Analysis” [Mesh] OR “Pragmatic Clinical Trials as Topic” [Mesh] OR “Clinical Trials, Phase II” [Publication Type] OR “Clinical Trials, Phase III” [Publication Type] OR “Clinical Trials, Phase IV” [Publication Type] OR “Controlled Clinical Trials” [Publication Type] OR “Randomized Controlled Trials” [Publication Type] OR “Pragmatic Clinical Trials as Topic” [Publication Type] OR “Single-Blind Method” [Mesh] OR “Double-Blind Method” [Mesh] OR random∗ [Title/Abstract] OR blind∗ [Title/Abstract] OR singleblind∗ [Title/Abstract] OR doubleblind∗ [Title/Abstract] OR trebleblind∗ [Title/Abstract] OR tripleblind∗ [Title/Abstract]
#4	#1 AND #2 AND #3

### 2.3 Eligibility criteria

#### 2.3.1 Population

Breast cancer patients over 18 years old confirmed by pathology or by cytology received treatment, such as surgery, chemotherapy, radiotherapy, and hormone therapy, regardless of region, race, and education.

#### 2.3.2 Intervention

Intervention is that using Baduanjin in rehabilitation, whether or not combined with routine drug therapy. There are no restrictions on the type, frequency, length, and stage.

#### 2.3.3 Control

The control group can be any other treatments, such as physical training in other forms, conventional nursing measures, psychological therapy, routine activities, and no treatment.

#### 2.3.4 Outcomes

The outcomes can be quality of postoperation life, emotions (depression, anxiety), or physical rehabilitation (body mass index, heart rate variability, lung capacity, arm circumference, shoulder range of motion, step test index).

#### 2.3.5 Study type

Studies must be randomized controlled trials that assessed the effects of Baduanjin on breast cancer patients. Reviews, conference proceedings, case reports, observational studies, non-random studies will be excluded.

#### 2.3.6 Characteristic

Only the full text will be included, no matter what the language is.

### 2.4 Study selection

**Figure 1:**
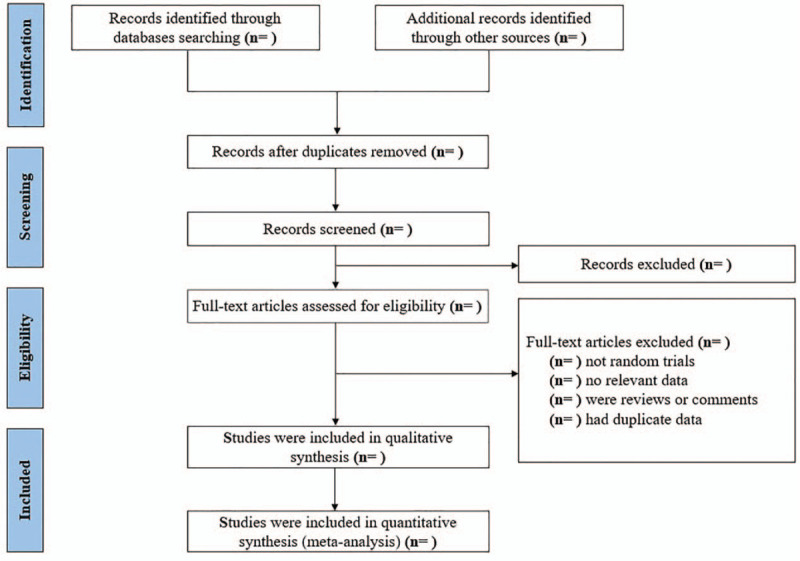
Flow diagram of study selection process.

All retrieved articles will be imported into reference management software (EndNote). Then, remove repetitive articles. Two researchers will independently screen the titles and abstracts according to eligibility criteria. The studies will be grouped into included studies, excluded studies and undetermined classified studies. We will record all the excluded reasons. If there are opinions that arise any conflict between 2 researchers, we will resolve it by conference, or by the third reviewer. The study selection process will be presented in a flow diagram (Fig. 1).

### 2.5 Data extraction

Two independent researchers will extract data according to the following items: author, year, funding, number of patients, publication time, study period, grouping and sample size, age and sex of patients, intervention method, basic treatments, whether to adopt the blindness, outcomes of interest, and follow-up time. We will utilize the formal data extract sheets in Excel to execute the program. When these items arise disagreement, we will solve it through the third reviewer.

### 2.6 Risk of bias

The risk of bias assessment will be implemented by 2 trained reviewers independently. We will use the “Cochrane bias risk assessment tool”.^[18]^ The assessment list including the following 7 items:

1. Generation of random sequence (selection bias).
2. Allocation concealment (selection bias).
3. Blinding of participants and researchers (performance bias).
4. Blinding of outcome assessment (detection bias).
5. Integrity outcome data (attrition bias).
6. Selective reporting (reporting bias).
7. Other sources of bias (other bias).

Each item will be classified as low risk, high risk, and unclear risk. Two experienced researchers will accomplish this job. Contradictory will reach agreements through negotiation or by the third researcher.

### 2.7 Statistical analysis

#### 2.7.1 Data synthesis

Statistical analysis will be performed with software Stata (13.0; Stata Corporation, College Station, TX). We will conduct meta-analyses using the inverse variance method to compute relative risks and their 95% confidence interval (CI) for dichotomous outcomes and mean difference and 95% CI for continuous outcomes. The statistical level of significance will be set at *P* < .05.

#### 2.7.2 Assessment of heterogeneity

Following the standard, we will use the Q test (Cochrane Q) and *I*^2^ to evaluate heterogeneity in our research. *P* ≥ .05 or *I*^2^ ≤ 50%, indicates that there is no significant statistical difference, and we will pool data with the fixed-effects model. If *P* < .05 (Q test) or *I*^2^ > 50%, we will use the random-effects model for the presence of heterogeneity. We will explore the heterogeneity by subgroup analysis and sensitivity analysis.

#### 2.7.3 Subgroup analysis and sensitivity analysis

While heterogeneity exists among studies, we will utilize subgroup analysis or sensitivity analysis. Subgroup analysis will be conducted on different sectionalization (age, operational styles, outcomes, whether Baduanjin unites with regular medical therapy) to explore the source of heterogeneity. Sensitivity analysis, differing from the subgroup analysis, can locate the low-quality studies in meta-analysis. The analysis will replicate many times by eliminating 1 study each time. For each analysis, we will get an overall effect value and 95% CI. If the data changes little, we can confirm that the result of the meta-analysis is reliable.

#### 2.7.4 Publication bias

We will use the funnel plot and Egger test to assess potential publication bias for outcomes with studies no less than 10.

### 2.8 Certainty of evidence

The certainty of evidence for each meta-analysis will be evaluated using the Grading of Recommendations Assessment, Development and Evaluation (GRADE) framework, which rated the quality considering 5 criteria: risk of bias, inconsistency, imprecision, indirectness, and publication bias.^[19]^ The quality of evidence will be rated as high, moderate, low, or very low.

## 3 Discussion

Currently, breast cancer patients in rehabilitation will be increasingly concerned by researchers. The randomized controlled trials about the effectiveness of Baduanjin have been proceeding well with recovering breast cancer patients. Many advantages were discovered, such as improving quality of life, altering the depression of patients.^[16]^ Of course, there are more functions of Baduanjin in recovery breast cancer patients for us to explore. However, whether the exercise is effective, which aspects can be improved by Baduanjin, there is still no certain answer. Thus, we must create more convincing evidence according to the existing trials. This systematic review is designed to supply strong evidence for guiding clinical work.

## Author contributions

**Conceptualization:** Yuqin Lin, Bin Pu.

**Formal analysis:** Guangyan Chen, Yuqin Lin, Bin Pu.

**Funding acquisition:** Xiyun Zhao.

**Investigation:** Guangyan Chen, Yuqin Lin, Xiyun Zhao, Bin Pu.

**Methodology:** Guangyan Chen, Bin Pu.

**Project administration:** Bin Pu.

**Resources:** Guangyan Chen, Xiyun Zhao.

**Software:** Guangyan Chen.

**Supervision:** Bin Pu.

**Validation:** Bin Pu.

**Visualization:** Guangyan Chen, Yuqin Lin, Bin Pu.

**Writing – original draft:** Guangyan Chen, Yuqin Lin, Bin Pu.

**Writing – review & editing:** Guangyan Chen, Bin Pu.

## References

[1] Siegel RL Miller KD Jemal A. Cancer statistics, 2020. CA Cancer J Clin 2020;70:07–30. 10.3322/caac.21590 31912902

[2] Siegel RL Miller KD Jemal A. Cancer statistics, 2019. CA Cancer J Clin 2019;69:07–34. 10.3322/caac.21551 30620402

[3] Chen W Zheng R Baade PD. Cancer statistics in China, 2015. CA Cancer J Clin 2016;66:115–32. 26808342 10.3322/caac.21338

[4] Sun YS Zhao Z Yang ZN. Risk factors and preventions of breast cancer. Int J Biol Sci 2017;13:1387–97. 29209143 10.7150/ijbs.21635 PMC5715522

[5] DeSantis CE Ma J Goding Sauer A. Breast cancer statistics, 2017, racial disparity in mortality by state. CA Cancer J Clin 2017;67:439–48. 28972651 10.3322/caac.21412

[6] Akram M Iqbal M Daniyal M. Awareness and current knowledge of breast cancer. Biol Res 2017;50:33. 28969709 10.1186/s40659-017-0140-9 PMC5625777

[7] Egan MY McEwen S Sikora L. Rehabilitation following cancer treatment. Disabil Rehabil 2013;35:2245–58. 23488617 10.3109/09638288.2013.774441

[8] Koh TC. Baduanjin -- an ancient Chinese exercise. Am J Chin Med 1982;10:14–21. 7183203 10.1142/S0192415X8200004X

[9] Li R Jin L Hong P. The effect of baduanjin on promoting the physical fitness and health of adults. Evid Based Complement Alternat Med 2014;2014:784059. 25050127 10.1155/2014/784059 PMC4094699

[10] Li H Ge D Liu S. Baduanjin exercise for low back pain: a systematic review and meta-analysis. Complement Ther Med 2019;43:109–16. 30935517 10.1016/j.ctim.2019.01.021

[11] Zou L Yeung A Quan X. A systematic review and meta-analysis of mindfulness-based (Baduanjin) exercise for alleviating musculoskeletal pain and improving sleep quality in people with chronic diseases. Int J Environ Res Public Health 2018;15:206. 10.3390/ijerph15020206 PMC5858275 29370149

[12] Cheng FK. Effects of Baduanjin on mental health: a comprehensive review. J Bodyw Mov Ther 2015;19:138–49. 25603754 10.1016/j.jbmt.2014.11.001

[13] Chen M Ou L Chen Y. Effectiveness and safety of Baduanjin exercise (BDJE) on heart failure with preserved left ventricular ejection fraction (HFpEF): a protocol for systematic review and meta-analysis. Medicine (Baltimore) 2020;99:e22994. 33181663 10.1097/MD.0000000000022994 PMC7668503

[14] Li J Yin S Li R. Baduanjin exercise for patients with knee osteoarthritis: a protocol for systematic review and meta-analysis. Medicine (Baltimore) 2020;99:e22963. 33126367 10.1097/MD.0000000000022963 PMC7598779

[15] Ma Q Li H Gao Y. Effects of Baduanjin on glucose and lipid metabolism in diabetic patients: a protocol for systematic review and meta-analysis. Medicine (Baltimore) 2021;100:e23532. 33530160 10.1097/MD.0000000000023532 PMC7850686

[16] Ying W Min QW Lei T. The health effects of Baduanjin exercise (a type of Qigong exercise) in breast cancer survivors: a randomized, controlled, single-blinded trial. Eur J Oncol Nurs 2019;39:90–7. 30850143 10.1016/j.ejon.2019.01.007

[17] Moher D Shamseer L Clarke M. Preferred reporting items for systematic review and meta-analysis protocols (PRISMA-P) 2015 statement. Syst Rev 2015;4:01. 10.1186/2046-4053-4-1 PMC4320440 25554246

[18] Higgins JPT Altman DG Sterne JAC. Chapter 8: assessing risk of bias in included studies Higgins JPT, Green S. Cochrane Handbook for Systematic Reviews of Interventions Version 5. 1. 0. Cochrane Collaboration 2011.

[19] Guyatt GH Oxman AD Vist GE. GRADE: an emerging consensus on rating quality of evidence and strength of recommendations. BMJ (Clinical research ed) 2008;336:924–6. 10.1136/bmj.39489.470347.AD PMC2335261 18436948

